# Diagnosis of Brain Diseases via Multi-Scale Time-Series Model

**DOI:** 10.3389/fnins.2019.00197

**Published:** 2019-03-14

**Authors:** Zehua Zhang, Junhai Xu, Jijun Tang, Quan Zou, Fei Guo

**Affiliations:** ^1^School of Computer Science and Technology, College of Intelligence and Computing, Tianjin University, Tianjin, China; ^2^School of Artificial Intelligence, College of Intelligence and Computing, Tianjin University, Tianjin, China; ^3^Department of Computer Science and Engineering, University of South Carolina, Columbia, SC, United States; ^4^Institute of Fundamental and Frontier Sciences, University of Electronic Science and Technology of China, Chengdu, China

**Keywords:** functional magnetic resonance imaging, multi-scale time-series, Alzheimer's disease, major depressive disorder, functional connection

## Abstract

The functional magnetic resonance imaging (fMRI) data and brain network analysis have been widely applied to automated diagnosis of neural diseases or brain diseases. The fMRI time series data not only contains specific numerical information, but also involves rich dynamic temporal information, those previous graph theory approaches focus on local topology structure and lose contextual information and global fluctuation information. Here, we propose a novel multi-scale functional connectivity for identifying the brain disease via fMRI data. We calculate the discrete probability distribution of co-activity between different brain regions with various intervals. Also, we consider nonsynchronous information under different time dimensions, for analyzing the contextual information in the fMRI data. Therefore, our proposed method can be applied to more disease diagnosis and other fMRI data, particularly automated diagnosis of neural diseases or brain diseases. Finally, we adopt Support Vector Machine (SVM) on our proposed time-series features, which can be applied to do the brain disease classification and even deal with all time-series data. Experimental results verify the effectiveness of our proposed method compared with other outstanding approaches on Alzheimer's Disease Neuroimaging Initiative (ADNI) dataset and Major Depressive Disorder (MDD) dataset. Therefore, we provide an efficient system via a novel perspective to study brain networks.

## 1. Introduction

The functional Magnetic Resonance Imaging (fMRI) technique provides an opportunity to quantify functional integration via measuring the correlation between intrinsic Blood-Oxygen-Level-Dependent (BOLD) signal fluctuations of distributed brain regions at rest. The BOLD signal is sensitive to spontaneous neural activity within brain regions, thus it can be used as an efficient and noninvasive way for investigating neurological disorders at the whole-brain level. Functional connectivity (FC), defined as the temporal correlation of BOLD signals in different brain regions, can exhibit how structurally segregated and functionally specialized brain regions interact with each other. Therefore, the brain network analysis using fMRI data will provide great advantages to automated diagnosis of neural diseases or brain diseases.

Some researchers model the FC information as a specific network by using graph theoretic techniques. Differences between normal and disrupted FC networks caused by pathological attacks provide important biomarkers to understand pathological underpinnings, in terms of the topological structure and connection strength. The network analysis has been becoming an increasingly useful tool for understanding the cerebral working mechanism and mining sensitive biomarkers for neural or mental diseases. Zeng et al. (2018) propose a new switching delayed particle swarm optimization (SDPSO) algorithm is proposed to optimize the SVM parameters. Using graph theories, the brain network analysis provides an effective solution to concisely quantify the connectivity properties of brain networks, where each node denotes a particular anatomical element or a brain region, and each edge represents the relationship between a pair of nodes, such as anatomical, functional or effective connections (Friston, 2011). The anatomical connection typically corresponds to white matter tracts between many pairs of brain regions. The functional connection corresponds to magnitudes of temporal correlations in activity and occurs between some pairs of anatomically unconnected regions, which may reflect linear or nonlinear interactions, as well as interactions within different time scales (Zhou et al., 2009). The effective connection represents direct or indirect causal influences of one region on another region, which may be estimated from observed perturbations whether synchronous or asynchronous (Friston et al., 2003). As a brain network analysis approach, the graph theory offers two important advantages (Tijms et al., 2013). One is that it provides quantitative measurement, which can preserve the connectivity information in the network and thus reflect the segregated and integrated nature of local brain activity. The other is that, it provides a general framework for comparing heterogeneous graphs constructed by different types of data, such as anatomical and functional data.

However, these graph theory approaches have many drawbacks that must be overcome. First, the graph theory has many limitations, on the one hand, common graph theory features such as edge weights, path lengths and clustering coefficients (Rubinov and Sporns, 2010; Chen et al., 2011) usually focus on local topology structure and lose their global topology characteristics (Sanz-Arigita et al., 2010; Jie et al., 2018); on the other hand, each node in the brain networks is uniquely corresponding to a specific brain region, mostly ignoring the label information of each node (Jie et al., 2018). Second. the functional connectivity is more sensitive to local information rather than the global topology, but some recent studies (Hutchison et al., 2013; Leonardi et al., 2013; Zeng et al., 2013, 2014; Allen et al., 2014) indicate that the FC network contains rich dynamic temporal information. To be more concrete, for each brain region, a sliding window approach is performed to generate a set of BOLD subseries on schizophrenia disease diagnosis (Damaraju et al., 2014) and others (Chen et al., 2016; Wee et al., 2016). Third, the raw functional data is underutilized, building brain network from raw data may lose the temporal or context information. For example, Pearson's Correlation Coefficient (PCC) is the simplest and most commonly scheme in functional connectivity estimation, which is the covariance of the two variables divided by the product of their standard deviations. Clearly, according to the mathematical definition, the PCC value is context-independent or order-independent in time series, not considering nonsynchronous information under different time dimensions.

**Figure 1:**
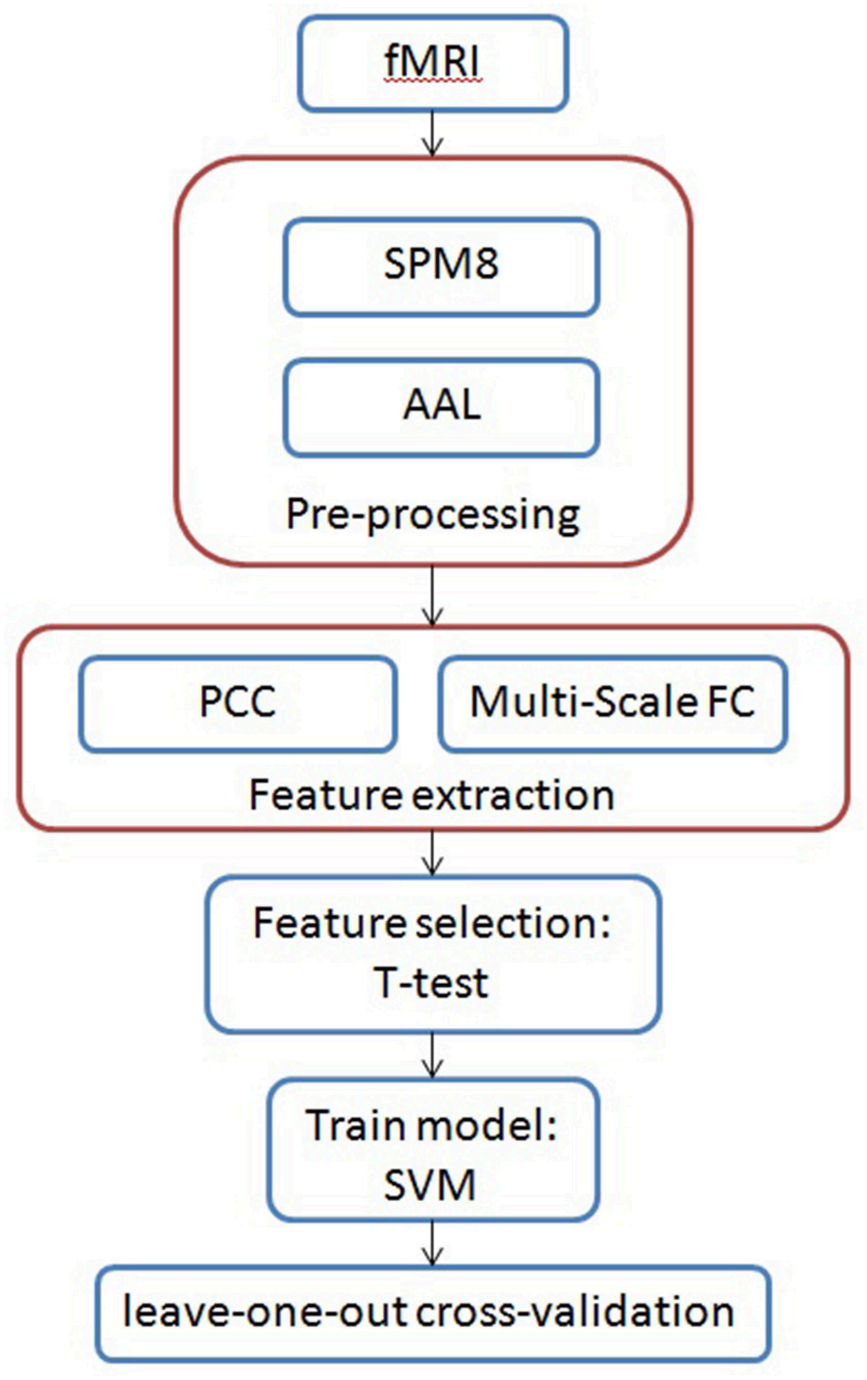
The framework of our proposed multi-scale time-series model for brain diseases diagnosis.

In view of the above, the fMRI time series not only contains specific numerical information, but also involves contextual information and global fluctuation information. In this paper, we propose a novel time-series model based on Jensen-Shannon divergence for identifying the brain disease via fMRI data, and the flow chart is shown in Figure 1. First, we calculate the discrete probability distribution of co-activity between different brain regions with various intervals in multi-scale time series data. Second, the contextual information is taken into account in analyzing the correlation and causality among the fMRI data. Third, we design a novel method based on time-series to measure the similarity between two object co-activity intensity of brain functional connectivity. Finally, we adopt Support Vector Machine (SVM) on our proposed time-series features, which can be applied to do the brain disease classification and even deal with all time-series data. Experimental results verify the effectiveness of our proposed method compared with other outstanding approaches on Alzheimer's Disease Neuroimaging Initiative (ADNI) dataset and Major Depressive Disorder (MDD) dataset. The rest of this paper is organized as follows. We start by a brief review of dataset and pre-processing. Then, we formulate the problem and present our proposed method. Finally, experimental results are reported, followed by the conclusion of this work.

## 2. Materials and Methods

In this section, we introduce the flow of our method. First, we preprocessed the original data, removed the noise from the original data, and segmented the fMRI image data through the brain region template. Next, we extract information or features from the perspective of functional connection between brain regions. To overcome the shortcoming of traditional Pearson Correlation Coefficient (PCC) methods, we propose a novel framework for feature extraction of brain functional connection. Then, through feature selection, we use the classification model for predicting brain disease. Finally, we discuss parameter settings in the model.

### 2.1. Dataset

We carry out experiments on two different datasets. One is a public Alzheimer's Disease Neuroimaging Initiative database (Jack et al., 2010), and another one is a volunteer experiment of Major Depressive Disorder (Geng et al., 2018). In the data pre-processing, we deal with the raw data by a widely used software package (SPM12), and then divide one brain into 116 brain regions.

#### 2.1.1. ADNI

In Alzheimer's Disease Neuroimaging Initiative database, we emply a total of 169 subjects, including 87 Alzheimer's patients (49 females and 38 males) and 82 normal controls (46 females and 36 males). We download the ADNI data from website http://adni.loni.usc.edu/.

#### 2.1.2. MDD

In volunteer experiment, we use a total of 60 subjects, including 31 volunteers with Major Depressive Disorder (MDD) (22 females and 8 males, aged 60.5 ± 11.2 years, range 25 − 65 years) and 29 healthy volunteers (18 females and 11 males, aged 50.1 ± 10.6 years, range 25 − 65 years). Those major depressive disorder subjects without comorbidity had a minimum duration of illness more than 3 months. Each participant provided written informed consent and the study was conducted in accordance with the local Ethics Committee.

### 2.2. Pre-processing

We perform image pre-processing for the fMRI data using a standard pipeline, carried out via the statistical parametric mapping (SPM12, www.fil.ion.ucl.ac.uk/spm/software/spm12/) software package on Matlab. The data pre-processing procedure includes slice timing, realign, segment, normalization and band-pass filtered. For more detailed data pre-processing procedure, please refer to website.

The whole brain of each subject in fMRI space is parcellated into 116 brain regions of interest (ROI) according to the Automated Anatomical Labeling (AAL) template. This atlas divided the brain into 78 cortical regions, 26 cerebellar regions and 12 subcortical regions according to anatomy, details in literature (Tzourio-Mazoyer et al., 2002). For each of the 116 ROIs, the mean time series was calculated by averaging the Blood-Oxygen-Level-Dependent (BOLD) signals among all voxels within the specifically ROI. There exist many similar templates such as Brainnetome template (Fan et al., 2016) and Harvard-Oxford template.

### 2.3. Feature Extraction

After pre-processing, how to excavate the location and cause of lesions is the focus of our research and attention. The most common method is to calculate the correlation between two brain regions through Pearson Correlation Coefficient (PCC), and analyze lesions by observing the changes of correlation. However, the PCC value is context-independent or order-independent, that is not considering nonsynchronous information at different time intervals. Here, we first give a basic introduction to PCC, and then elaborate on our approach.

#### 2.3.1. Pearson Correlation Coefficient

Pearson's correlation coefficient (PCC) is the simplest and most commonly scheme in functional connectivity estimation. For any two brain regions, the coordination degree of blood-oxygen-level dependent fluctuation is calculated as the functional connection strength between these two brain regions. Typically, in the case of the AAL template, this step extracts the 6,670-dimensional features. Mathematical definition is the covariance of the two variables divided by the product of their standard deviations, as follows:

(1) $$\begin{array}{l} PCC_{X,Y}=\frac{\text{E}\left[\left(X-\mu_{X}\right)\left(Y-\mu_{Y}\right)\right]}{\sigma_{X}\sigma_{Y}} \end{array}$$

Clearly, according to the formula, the value of the Pearson's correlation coefficient is context-independent or order-independent in time series, which it only limits alignment at the same time, so information about the time dimension or context is missing.

#### 2.3.2. Multi-Scale Functional Connectivity of Brain Regions

We extract the discrete probability distribution of co-activity in time series data. First, we use the function *ϕ*(·) to evaluate temporal dynamic property of the time series data. In addition, we convert *ϕ*(·) to *g*(·), defined as follows:

(2) $$\begin{array}{l} \phi \left(t_{i_{1},j_{1}}^{k_{1}},t_{i_{2},j_{2}}^{k_{2}}\right)=g\left(f_{\varphi }\left(t_{i_{1},j_{1}}^{k_{1}}\right),f_{\varphi }\left(t_{i_{2},j_{2}}^{k_{2}}\right)\right) \end{array}$$

where *f*(·) represents a mapping function that makes use of prior knowledge in order to map the original time series into another specific form, *g*(·) represents the function to evaluate temporal information after the mapping operation.

We utilize the prior knowledge in order to map the original multivariate time series data into another specific form, such as a mapping of numeric, state and character. The mapping function is defined as follows:

(3) $$\begin{array}{l} f_{\varphi }(A_{k})=f_{\varphi }\{T_{1}^{k},T_{2}^{k},\cdots ,T_{i}^{k},\cdots ,T_{N}^{k}\}\\ \;=\{f_{\varphi }(T_{1}^{k}),f_{\varphi }(T_{2}^{k}),\cdots ,f_{\varphi }(T_{N}^{k})\} \end{array}$$

where *A*_*k*_ denotes the original time series data, and *φ* denotes the prior knowledge.

In the multivariate time series data *A*_*k*_, the correlation value between $T_{i}^{k}$ and $T_{j}^{k}$ is defined as follows:

(4) $$\begin{array}{l} C_{\phi \left(\cdot \right)}^{k}\left(i,j\right)=\overset{M}{\underset{m\;=\;1}{\sum }}\phi \left(t_{i,m}^{k},t_{j,m}^{k}\right) \end{array}$$

In addition, the correlation value between $T_{i}^{k}$ and $T_{j}^{k}$ in interval *I*_*t*_ = [*r*_*t*_, *s*_*t*_] is defined as follows:

(5) $$\begin{array}{l} C_{\phi \left(\cdot \right)}^{k}\left(i,j,I_{t}\right)=\overset{M}{\underset{m=1}{\sum }}\overset{s_{t}}{\underset{l=r_{t}}{\sum }}\phi \left(t_{i,m}^{k},t_{j,m+l}^{k}\right) \end{array}$$

Notably, it is obvious that $C_{\phi \left(\cdot \right)}^{k}\left(i,j,I_{t}\right)\neq C_{\phi \left(\cdot \right)}^{k}\left(j,i,I_{t}\right)$.

Generally, we explore the correlation of time series data in multiple intervals. Let $C_{\phi \left(\cdot \right)}^{k}\in R^{N\times N\times T}$ denotes the multi-scale weighted correlation coefficient in multivariate time series data *A*_*k*_. Here, $C_{\phi \left(\cdot \right)}^{k}$ is a 3-order tensor, *N* is the number of time series data, *T* is the number of intervals.

Next, we transform the tensor $C_{\phi \left(\cdot \right)}^{k}$ into a discrete probability distribution $P_{\phi \left(\cdot \right)}^{k}$ for analyzing co-activity in multi-scale time series data, as follows:

(6) $$\begin{array}{l} P_{\phi (\cdot )}^{k}=\{p_{\phi (\cdot )}^{k}(i,j,I_{t})|i,j\in [1,N],I_{t}\in I\} \end{array}$$

where $p_{\phi \left(\cdot \right)}^{k}\left(i,j,I_{t}\right)$ represents the proportion of correlation value between *i*-th time series data and *j*-th time series data based on function *ϕ*(·) in interval *I*_*t*_, defined as follows:

(7) $$\begin{array}{l} p_{\phi \left(\cdot \right)}^{k}\left(i,j,I_{t}\right)=\frac{C_{\phi \left(\cdot \right)}^{k}\left(i,j,I_{t}\right)}{\sum_{i=1}^{N}\sum_{j=1}^{N}\sum_{t=1}^{T}C_{\phi \left(\cdot \right)}^{k}\left(i,j,I_{t}\right)} \end{array}$$

### 2.4. Classification Model for Predicting Brain Disease

In disease prediction, the number of samples is limited, but the feature dimension is usually large, so we need to both compress the feature space to improve the accuracy and analyze the etiology with more meaningful features. We use *t*-test for feature selection, and then we use Support Vector Machine (SVM) as the learning model, which is described in detail as follows.

#### 2.4.1. Feature Selection

We use the two-sample *t*-test as the feature selection method. We assume that one feature of positive and negative samples is subject to the distribution of the same mean, and we set the significance parameter *p* = 0.05.

#### 2.4.2. Support Vector Machine

We adopt Support Vector Machine (SVM) technique developed by Cortes and Vapnik (1995) for solve the binary classification problem. Also, various kinds of binary classification model can be applied in many other biomedical prediction problems (Guo et al., 2014, 2015, 2016; Ding et al., 2016a,b, 2017a,b; Liu et al., 2016; Zeng et al., 2016; Shen et al., 2017a,b; Xuan et al., 2017; Pan et al., 2018). The decision function is shown as follows:

(8) $$\begin{array}{l} \gamma \left(A_{k}\right)=sign\left\{\overset{K}{\underset{i=1}{\sum }}\alpha_{i}y_{i}\cdot K\left(A_{k},A_{i}\right)+b\right\} \end{array}$$

where $K\left(A_{k},A_{i}\right)$ represents our proposed novel time-series kernel function, and α_*i*_ is calculated as follows:

(9) $$\begin{array}{l} \text{Maximize }\sum_{i=1}^{K}\alpha_{i}-\frac{1}{2}\sum_{i=1}^{K}\sum_{j=1}^{K}\alpha_{i}\alpha_{j}\cdot y_{i}y_{j}\cdot K(A_{i},A_{j})\\ \text{ s.t. }0\leq \alpha_{i}\leq C\\ \;\sum_{i=1}^{K}\alpha_{i}\gamma_{i}=0 \end{array}$$

where *C* is a regularization parameter that controls the tradeoff between margin and misclassification error.

### 2.5. Model Parameter

In practice, we make more detailed discussion for parameters in our method. We discuss some prior knowledge and assumptions in our problem of Alzheimer's disease and Major Depression Disorder diagnosis, and some details need to be clarified. The time series data not only carry specific numerical information, but also include contextual and fluctuation trend information.

Here, due to the BOLD imaging principle, we pay more attention to the time points of high activity state, that is, time points with high values in time series. We define a dynamic or soft threshold to distinguish whether a time point is active or not, that is, converting a numeric sequence into a state sequence or 0/1 sequence.

For all active time points in one set of time series, we count the number of time points of simultaneous responses in other sets of time series. Moreover, we analyze the co-active between two sets of time series in asynchronous. As we get more details with asynchronous analysis, we'll get more essential information. In the experiments, it is also proved by the higher classification accuracy.

#### 2.5.1. Time Series Mapping

We adopt a empirical rule to indicate the dynamic threshold, called three-sigma method (WalterA, 1986). This method converts a numeric sequence into a state sequence, the dynamic threshold represented as follows:

(10) $$\begin{array}{l} th\left(T_{i}^{k}\right)=\mu \left(T_{i}^{k}\right)+\eta \cdot \sigma \left(T_{i}^{k}\right) \end{array}$$

where

(11) $$\begin{array}{l} \mu \left(T_{i}^{k}\right)=\frac{\sum_{m=1}^{M}t_{i,m}^{k}}{\left|T_{i}^{k}\right|} \end{array}$$

and

(12) $$\begin{array}{l} \sigma \left(T_{i}^{k}\right)=\frac{\sum_{m=1}^{M}{\left(t_{i,m}^{k}-\mu \left(T_{i}^{k}\right)\right)}^{2}}{|T_{i}^{k}|-1} \end{array}$$

In a multivariate time series *A*_*k*_, we calculate a corresponding dynamic threshold $th\left(T_{i}^{k}\right)$ for each set of time series $T_{i}^{k}$. Then, for a set of time series $T_{i}^{k}$, we convert a numeric sequence into a 0/1 sequence according to mapping function *f*(·), as follows:

(13) $$\begin{array}{l} f(t_{i,m}^{k})=\{\begin{array}{ll} 1, & t_{i,m}^{k}\geq th(T_{i}^{k})\\ 0, & \text{else} \end{array} \end{array}$$

The magnitude of η indicates the sensitivity of our method to the active state. In our experiment, η is set to 1.

#### 2.5.2. Correlation Function *ϕ*

The correlation function represents the relationship between a couple of time points in time series. In disease diagnosis, we only focus on co-activity, that is, both brain region *i* in time point *m* and brain region *j* in time point *n* are in active states. To be more concrete, $t_{i,m}^{k}$ and $t_{j,n}^{k}$ are greater than the threshold $th\left(T_{i}^{k}\right)$ and $th\left(T_{j}^{k}\right)$, respectively.

(14) $$\begin{array}{l} g(f(t_{i,m}^{k}),f(t_{j,n}^{k}))=\{\begin{array}{ll} 1, & \text{f}(t_{i,m}^{k})=f(t_{j,n}^{k})=1\\ 0, & \text{else} \end{array} \end{array}$$

Corresponding to Formula 2 above, *ϕ*(·) in our experiment is:

(15) $$\begin{array}{l} \phi (t_{i,m}^{k},t_{j,n}^{k})=\{\begin{array}{ll} 1, & t_{i,m}^{k}\geq th(T_{i}^{k})\&t_{j,n}^{k}\geq th(T_{j}^{k})\\ 0, & \text{else} \end{array} \end{array}$$

#### 2.5.3. Interval Set *I*

For a collection of multiple intervals *I*, we extract local information by the element of interval, that is, greater element, more detailed information. Easy to be over-fit and sparse; if the element of interval is little, we may lose some key information. Also, for a interval *I*_*t*_ ∈ *I*, if *I*_*t*_ is close to zero, it means that two time points that we're interested in are very close; if *I*_*t*_ is far from zero, it indicates that we extract long-distance asynchronous information.

In our experiments, the interval collection *I* is set to {[0, 0], [1, 1], [2, 2], [3, 12]}. Here, [0, 0] represents information for synchronization, [1, 1] and [2, 2] represent short-distance correlation for asynchronism, [3, 12] represents a loose interval for asynchronism. Empirically, it is sensitive to close interval of zero and loose for long distances.

## 3. Results

Our experiment consists of three parts. To proof the effectiveness of our approach, we perform on automated diagnoses of Alzheimer's disease and Major Depressive Disorder, respectively. We evaluate the classification performance using the leave-one-out cross-validation (LOOCV). And also, we adopt Accuracy, Sensitivity, Specificity and AUC as evaluation standards. First, we compare the results of the traditional PCC method and our feature extraction method in the two data sets of AD and MDD. Then, we compare the effects of different classifiers. Finally, we compare our approach with some recent research works.

### 3.1. Comparison of Different Features

Here, we compare the performance of traditional PCC method and our feature extraction method to analyze fMRI data. In addition to feature extraction, we use the same experimental steps and parameters, including preprocessing, feature selection and classifier. The results are shown in Table 1.

**Table 1:** Comparison of different features.

**Method**	**Data set**	**Accuracy**	**Sensitivity**	**Specificity**	**AUC**
PCC	AD	0.5858	0.5747	0.5976	0.5612
Multi-Scale FC	AD	0.8876	0.8506	0.9268	0.8562
Multi-Scale FC + PCC	AD	0.8935	0.8850	0.9024	0.8748
PCC	MDD	0.6167	0.6129	0.6207	0.6514
Multi-Scale FC	MDD	0.9000	0.8710	0.9310	0.9295
Multi-Scale FC + PCC	MDD	0.8667	0.8065	0.9310	0.8961

On Alzheimer's disease and major depressive disorder database, we compare our method to traditional PCC method, and classification results are summarized in Table 1. The information extracted by our multi-scale functional connection (Multi-Scale FC) method is used for predicting brain disease, which is obviously higher than the traditional PCC method. On Alzheimer's disease dataset, our method achieves best specificity of 0.9268. Moreover, by combining PCC and our method, we achieve better results, with ACC of 0.8935 and AUC of 0.8748. On MDD dataset, our method also achieve the best results, but the difference is that PCC and multi-scale functional connection are actually lower when combined. The experimental results indicate that our approach is more effective than traditional PCC or graph theory feature-based methods. Combining different methods will yield better results, but there is also a risk of over-fitting.

### 3.2. Comparison of Different Classifiers

In this part, we use the feature extraction model in the previous step to compare the performance of different classifiers. Specifically, we compare three classifiers: random forest (RF), logistic regression (LR) and support vector machine (SVM). The results are shown in Table 2.

**Table 2:** Comparison of different classifiers.

**Method**	**Data set**	**Accuracy**	**Sensitivity**	**Specificity**	**AUC**
LR	AD	0.8579	0.7931	0.9268	0.8347
RF	AD	0.8343	0.8276	0.8415	0.8284
SVM	AD	0.8935	0.8850	0.9024	0.8748
LR	MDD	0.8333	0.8387	0.8276	0.8684
RF	MDD	0.8833	0.9032	0.8621	0.8921
SVM	MDD	0.8667	0.8065	0.9310	0.8961

In this part, we use our proposed multi-scale functional connection method to extract features, and compare the results of different classifiers. Comparing these three classifiers, SVM can achieve the highest AUC in both AD dataset and MDD dataset, the best ACC can also be obtained on the AD data set, which is generally a stable classifier. In addition, RF can obtain the best ACC on the MDD dataset, and LR can obtain the best Spe on the AD dataset. Overall, all three classifiers can achieve good accuracy, indicating that the information extracted by our method is effective and stable.

### 3.3. Comparison of Different Existing Methods

We compare our proposed method to recent outstanding studies. Baseline represents the traditional graph theory feature-based method. Moreover, the state-of-the-art methods represent three major groups of graph kernels on edge, subtree and shortest-path, respectively. These graph kernel belong to the Weisfeiler-Lehman graph kernel framework (Shervashidze et al., 2011), denoted as WL-edge, WL-subtree and WL-shortestpath, respectively. In addition, in the Alzheimer's disease diagnosis, we also compare with the graph kernel method with shortest-path (Shortest-path) (Borgwardt and Kriegel, 2006), the sliding window method (FON: 70-length sliding window with 1-step) (Chen et al., 2016) and the sub-network kernel method (SKL) (Jie et al., 2018). In the Major Depressive Disorder classification problem, we compare to the method of Geng et al. (2018).

On Alzheimer's Disease Neuroimaging Initiative database, we compare our method to seven existing methods, and classification results are summarized in Table 3. Our method achieves best accuracy of 0.8876 and best AUC of 0.8562. However, the accuracy values for Baseline, FON, Shortest-path, WL-edge, WL-subtree, WL-Shortestpath and SKL are 0.5858, 0.8580, 0.7396, 0.6272, 0.7811, and 0.6095, respectively. Also, the AUC values for these seven methods are 0.5612, 0.8195, 0.6938, 0.6084, 0.7645, and 0.5735, respectively. Comparing to these methods, our method achieves accuracy improvement of 0.0296 and AUC improvement of 0.0367, respectively. The experimental results indicate that our approach is far better than traditional graph theory feature-based methods, and slightly better than the state-of-the-art graph kernel-based methods.

**Table 3:** Comparison of different existing methods on ADNI.

**Method**	**Accuracy**	**Sensitivity**	**Specificity**	**AUC**
Baseline	0.5858	0.5747	0.5976	0.5612
FON	0.8580	0.8161	0.9024	0.8195
Shortest-path	0.7396	0.8161	0.6585	0.6938
WL-edge	0.6272	0.6437	0.6098	0.6084
WL-subtree	0.7811	0.7816	0.7805	0.7645
WL-Shortestpath	0.6095	0.5977	0.6220	0.5735
SKL	0.8462	0.8046	0.8902	0.8166
Our method	0.8876	0.8506	0.9268	0.8562

On the volunteer experiments of Major Depressive Disorder, we compare our method to three existing methods, and classification results are summarized in Table 4. Our method achieves best accuracy of 0.9000 and best AUC of 0.9295. However, the accuracy values for Baseline, Shortest-path and method of Xu et al. are 0.6167, 0.7833, and 0.8667, respectively. Also, the AUC values for these three methods are 0.6514, 0.8135, and 0.9103, respectively. Comparing to these methods, our method achieves accuracy improvement of 0.0333 and AUC improvement of 0.0192, respectively. The experimental results indicate that our approach is far better than traditional graph methods, and slightly better than the current outstanding methods.

**Table 4:** Comparison of different existing methods on MDD.

**Method**	**Accuracy**	**Sensitivity**	**Specificity**	**AUC**
Baseline	0.6167	0.6129	0.6207	0.6514
Shortest-path	0.7833	0.8065	0.7586	0.8135
Xu et al.	0.8667	0.8710	0.8621	0.9103
Our Method	0.9000	0.8710	0.9310	0.9295

## 4. Conclusions

The fMRI time series data not only contains specific numerical information, but also involves rich dynamic temporal information. However, those previous graph theory approaches focus on local topology structure and lose contextual information and global fluctuation information. Here, we propose a novel multi-scale functional connectivity for identifying the brain disease via fMRI data. We calculate the discrete probability distribution of co-activity between different brain regions with various intervals. Also, we consider nonsynchronous information under different time dimensions, for analyzing the contextual information in the fMRI data. Therefore, our proposed method can be applied to more disease diagnosis and other fMRI data, particularly automated diagnosis of neural diseases or brain diseases. Experimental results verify the effectiveness of our proposed method, so we provide an efficient system via a novel perspective to study brain networks.In the future, parallel computing (Zou et al., 2017), computational intelligence (Xu et al., 2017; Zou et al., 2017) and neural networks (Song et al., 2018; Xu et al., 2018) can be considered with the growing of dataset.

## Data Availability

Publicly available datasets were analyzed in this study. This data can be found here: http://adni.loni.usc.edu/. The results and codes for this study can be found in the https://github.com/guofei-tju/Multi-Scale-FC-Frontier-in-NeuroSci.git.

## Author Contributions

FG and QZ conceived and designed the experiments. ZZ and JX performed the experiments and analyzed the data. FG and ZZ wrote the paper. FG and JT supervised the experiments and reviewed the manuscript.

### Conflict of Interest Statement

The authors declare that the research was conducted in the absence of any commercial or financial relationships that could be construed as a potential conflict of interest.

## References

[B1] Allen E. A. Damaraju E. Plis S. M. Erhardt E. B. Eichele T. Calhoun V. D. (2014). Tracking whole-brain connectivity dynamics in the resting state. Cereb. Cortex 24, 663–676. 10.1093/cercor/bhs352 23146964 PMC3920766

[B2] Borgwardt K. M. Kriegel H. P. (2006). Shortest-path kernels on graphs, in IEEE International Conference on Data Mining (Houston, TX), 74–81. 10.1109/ICDM.2005.132

[B3] Chen G. Ward B. D. Xie C. Li W. Wu Z. Jones J. L.. (2011). Classification of alzheimer disease, mild cognitive impairment, and normal cognitive status with large-scale network analysis based on resting-state functional MR imaging. Int. J. Med. Radiol. 259, 213–221. 10.1148/radiol.10100734 21248238 PMC3064820

[B4] Chen X. Zhang H. Gao Y. Wee C. Y. Li G. Shen D. (2016). High-order resting-state functional connectivity network for MCI classification. Hum. Brain Mapp. 37, 3282–3296. 10.1002/hbm.23240 27144538 PMC4980261

[B5] Cortes C. Vapnik V. (1995). Support-vector networks. Mach. Learn. 20, 273–297. 10.1007/BF00994018

[B6] Damaraju E. Allen E. A. Belger A. Ford J. M. Mcewen S. Mathalon D. H.. (2014). Dynamic functional connectivity analysis reveals transient states of dysconnectivity in schizophrenia. Neuroimage Clin. 5, 298–308. 10.1016/j.nicl.2014.07.003 25161896 PMC4141977

[B7] Ding Y. Tang J. Guo F. (2016a). Identification of protein-protein interactions via a novel matrix-based sequence representation model with amino acid contact information. Int. J. Mol. Sci. 17:1623. 10.3390/ijms17101623 27669239 PMC5085656

[B8] Ding Y. Tang J. Guo F. (2016b). Predicting protein-protein interactions via multivariate mutual information of protein sequences. BMC Bioinformatics 17:398. 10.1186/s12859-016-1253-9 27677692 PMC5039908

[B9] Ding Y. Tang J. Guo F. (2017a). Identification of drug-target interactions via multiple information integration. Inform. Sci. 418–419, 546–560. 10.1016/j.ins.2017.08.045

[B10] Ding Y. Tang J. Guo F. (2017b). Identification of protein-ligand binding sites by sequence information and ensemble classifier. J. Chem. Inform. Model. 57, 3149–3161. 10.1021/acs.jcim.7b00307 29125297

[B11] Fan L. Li H. Zhuo J. Yu Z. Wang J. Chen L.. (2016). The human brainnetome atlas: a new brain atlas based on connectional architecture. Cereb. Cortex 26, 3508–3526. 10.1093/cercor/bhw157 27230218 PMC4961028

[B12] Friston K. J. (2011). Functional and effective connectivity in neuroimaging: a synthesis. Brain Connect 1, 13–36. 10.1089/brain.2011.0008 22432952

[B13] Friston K. J. Harrison L. Penny W. (2003). Dynamic causal modelling. Neuroimage 19, 1273–1302. 10.1016/S1053-8119(03)00202-7 12948688

[B14] Geng X. Xu J. Liu B. Shi Y. (2018). Multivariate classification of major depressive disorder using the effective connectivity and functional connectivity. Front. Neurosci. 12:38. 10.3389/fnins.2018.00038 29515348 PMC5825897

[B15] Guo F. Ding Y. Li S. C. Shen C. Wang L. (2016). Protein-protein interface prediction based on hexagon structure similarity. Comput. Biol. Chem. 63, 83–88. 10.1016/j.compbiolchem.2016.02.008 26936323

[B16] Guo F. Ding Y. Li Z. Tang J. (2015). Identification of protein-protein interactions by detecting correlated mutation at the interface. J. Chem. Inform. Model. 55, 2042–2049. 10.1021/acs.jcim.5b00320 26284382

[B17] Guo F. Li S. C. Du P. Wang L. (2014). Probabilistic models for capturing more physicochemical properties on protein-protein interface. J. Chem. Inform. Model. 54, 1798–1809. 10.1021/ci5002372 24881460

[B18] Hutchison R. M. Womelsdorf T. Allen E. A. Bandettini P. A. Calhoun V. D. Corbetta M.. (2013). Dynamic functional connectivity: promise, issues, and interpretations. Neuroimage 80, 360–378. 10.1016/j.neuroimage.2013.05.079 23707587 PMC3807588

[B19] Jack C. R. Bernstein M. A. Fox N. C. Thompson P. Alexander G. Harvey D.. (2010). The Alzheimer's disease neuroimaging initiative (ADNI): MRI methods. J. Magn. Reson. Imaging 27, 685–691. 10.1002/jmri.21049 18302232 PMC2544629

[B20] Jie B. Liu M. Zhang D. Shen D. (2018). Sub-network kernels for measuring similarity of brain connectivity networks in disease diagnosis. IEEE Trans. Image Process. 27, 2340–2353. 10.1109/TIP.2018.2799706 29470170 PMC5844189

[B21] Leonardi N. Richiardi J. Gschwind M. Simioni S. Annoni J. M. Schluep M.. (2013). Principal components of functional connectivity: a new approach to study dynamic brain connectivity during rest. Neuroimage 83, 937–950. 10.1016/j.neuroimage.2013.07.019 23872496

[B22] Liu Y. Zeng X. He Z. Zou Q. (2016). Inferring microrna-disease associations by random walk on a heterogeneous network with multiple data sources. IEEE/ACM Trans. Comput. Biol. Bioinformatics 14, 905–915. 10.1109/TCBB.2016.2550432 27076459

[B23] Pan G. Jiang L. Tang J. Guo F. (2018). A novel computational method for detecting dna methylation sites with dna sequence information and physicochemical properties. Int. J. Mol. Sci. 19:E511. 10.3390/ijms19020511 29419752 PMC5855733

[B24] Rubinov M. Sporns O. (2010). Complex network measures of brain connectivity: uses and interpretations. Neuroimage 52, 1059–1069. 10.1016/j.neuroimage.2009.10.003 19819337

[B25] Sanz-Arigita E. J. Schoonheim M. M. Damoiseaux J. S. Rombouts S. A. R. B. Maris E. Barkhof F.. (2010). Loss of 'small-world' networks in Alzheimer's disease: graph analysis of fmri resting-state functional connectivity. PLoS ONE 5:e13788. 10.1371/journal.pone.0013788 21072180 PMC2967467

[B26] Shen C. Ding Y. Tang J. Song J. Guo F. (2017a). Identification of dna-protein binding sites through multi-scale local average blocks on sequence information. Molecules 22:2079. 10.3390/molecules22122079 29182548 PMC6149935

[B27] Shen C. Ding Y. Tang J. Xu X. Guo F. (2017b). An ameliorated prediction of drug-target interactions based on multi-scale discrete wavelet transform and network features. Int. J. Mol. Sci. 18:1781. 10.3390/ijms18081781 28813000 PMC5578170

[B28] Shervashidze N. Schweitzer P. Leeuwen E. J. V. Mehlhorn K. Borgwardt K. M. (2011). Weisfeiler-lehman graph kernels. J. Mach. Learn. Res. 12, 2539–2561. 10.1016/j.websem.2011.06.001

[B29] Song T. Liu X. Zeng X. (2018). Asynchronous spiking neural p systems with anti-spikes. IEEE Trans. Nanobiosci. 16, 888–895. 10.1007/s11063-014-9378-1

[B30] Tijms B. M. Wink A. M. De H. W. Wm V. D. F. Stam C. J. Scheltens P.. (2013). Alzheimer's disease: connecting findings from graph theoretical studies of brain networks. Neurobiol. Aging 34, 2023–2036. 10.1016/j.neurobiolaging.2013.02.020 23541878

[B31] Tzourio-Mazoyer N. Landeau B. Papathanassiou D. Crivello F. Etard O. Delcroix N.. (2002). Automated anatomical labeling of activations in spm using a macroscopic anatomical parcellation of the mni mri single-subject brain. Neuroimage 15, 273–289. 10.1006/nimg.2001.0978 11771995

[B32] WalterA (1986). Statistical Method From the Viewpoint of Quality Control. New York, NY: Dover.

[B33] Wee C. Y. Yang S. Yap P. T. Shen D. (2016). Sparse temporally dynamic resting-state functional connectivity networks for early mci identification. Brain Imaging Behav. 10, 342–356. 10.1007/s11682-015-9408-2 26123390 PMC4692725

[B34] Xu H. Zeng W. Zeng X. Yen G. G. (2018). An evolutionary algorithm based on minkowski distance for many-objective optimization. IEEE Trans. Cybern. 1–E12. Available online at: https://ieeexplore.ieee.org/document/8423202 10.1109/TCYB.2018.2856208 30059330

[B35] Xu H. Zeng W. Zhang D. Zeng X. (2017). MOEA/HD: a multiobjective evolutionary algorithm based on hierarchical decomposition. IEEE Trans. Cybern. 49, 517–526. 10.1109/TCYB.2017.2779450 29990272

[B36] Xuan Z. Quan Z. Rodruguez-Paton A. Zeng X. (2017). Meta-path methods for prioritizing candidate disease mirnas. IEEE/ACM Trans. Comput. Biol. Bioinformatics 16, 283–291. 10.1109/TCBB.2017.2776280 29990255

[B37] Zeng N. Qiu H. Wang Z. Liu W. Zhang H. Li Y. (2018). A new switching-delayed-pso-based optimized SVM algorithm for diagnosis of alzheimer's disease. Neurocomputing 320, 195–202. 10.1016/j.neucom.2018.09.001

[B38] Zeng N. Wang Z. Li Y. Du M. Cao J. Liu X. (2013). Time series modeling of nano-gold immunochromatographic assay via expectation maximization algorithm. IEEE Trans. Biomed. Eng. 60, 3418–3424. 10.1109/TBME.2013.2260160 23629840

[B39] Zeng N. Wang Z. Zineddin B. Li Y. Du M. Xiao L.. (2014). Image-based quantitative analysis of gold immunochromatographic strip via cellular neural network approach. IEEE Trans. Med. Imaging 33, 1129–1136. 10.1109/TMI.2014.2305394 24770917

[B40] Zeng X. Liao Y. Liu Y. Zou Q. (2016). Prediction and validation of disease genes using hetesim scores. IEEE/ACM Trans. Comput. Biol. Bioinformatics 14, 687–695. 10.1109/TCBB.2016.2520947 26890920

[B41] Zhou D. Thompson W. K. Siegle G. (2009). Matlab toolbox for functional connectivity. Neuroimage 47, 1590–1607. 10.1016/j.neuroimage.2009.05.089 19520177 PMC2728136

[B42] Zou Q. Wan S. Zeng X. Ma Z. S. (2017). Reconstructing evolutionary trees in parallel for massive sequences. BMC Syst. Biol. 11(Suppl. 6):100. 10.1186/s12918-017-0476-3 29297337 PMC5751538

